# Supplementary material for: Participation in a Voluntary Blood Donation Program as an Opportunity to Assess and Enhance Tetanus Immunity in Adult Blood Donors with an Outdated or Unknown Vaccination Status
Source: Vaccines (Basel). 2025 Aug 21;13(8):884. doi: 10.3390/vaccines13080884 (PMC12390347; doi:10.3390/vaccines13080884)
Supplement: Supplementary file 1 [file vaccines-13-00884-s001.zip › vaccines-3769346-supplementary.pdf]

QUESTIONNAIRE

We kindly ask you to answer the following questions. The survey is part of a research project entitled: „**Assessment of tetanus immunity in the Polish population aged over 50 years and the effectiveness of a single booster dose of dT vaccine in individuals who have not been vaccinated for over 30 years**".

1. First and last name: ……………………………………………………………………………………….

2. Sex: ☐ female ☐ male

3. Age: ………. years

4. Education: ☐ primary ☐ secondary ☐ higher

5. Are you currently employed? ☐ yes ☐ no

6. What is your current occupation?: ………………………………………………………………………..

7. Where do you live? ☐ urban area ☐ rural area

8. Have you been vaccinated against tetanus? ☐ yes ☐ no

9. Do you have documentation of your past vaccinations? ☐ yes ☐ no

10. Have you ever received additional doses of the tetanus vaccine? ☐ yes ☐ no

If yes, in which year and how many doses? (Please write):……………………………………………………………

11. Have you ever received an injection (vaccine) after an injury or accident? ☐ yes ☐ no

If yes, in which year and how many doses? (Please write):……………………………………………………………

12. Have you ever taken medications that suppress the immune system (e.g., chemotherapy, immunosuppressive drugs) or undergone radiotherapy? ☐ yes ☐ no

13. Have you ever had chronic diseases of the hematopoietic system or the kidneys? ☐ yes ☐ no

If yes, what kind and when? (Please write):…………………………………………………………………………...

14. Do you work in a garden with flowers or vegetables? ☐ yes ☐ no

15. Have you ever sustained an injury contaminated with soil? ☐ yes ☐ no

16. During your childhood, youth, or later in life, did you have daily contact with a rural farmyard environment where there were farm animals (horses, cows, pigs, or others)? ☐ yes ☐ no
